# Supplementary material for: Robust and inducible genome editing via an all-in-one prime editor in human pluripotent stem cells
Source: Nat Commun. 2024 Dec 30;15:10824. doi: 10.1038/s41467-024-55104-1 (PMC11685797; doi:10.1038/s41467-024-55104-1)
Supplement: Supplementary file 1 — Supplementary Information [file 41467_2024_55104_MOESM1_ESM.pdf]

Supplementary Figure.1

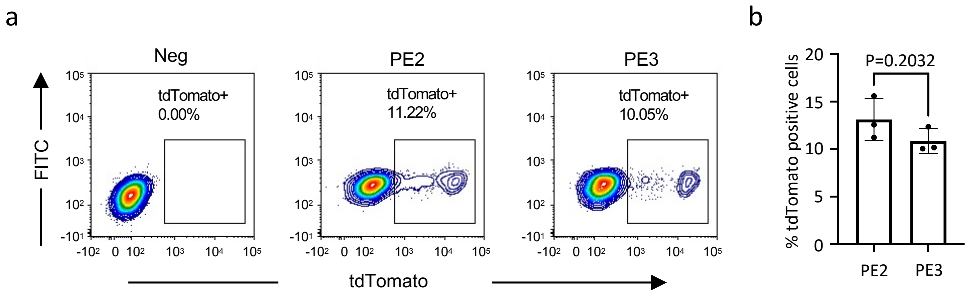

Supplementary Fig. 1: Flow cytometry analysis of the editing efficiency by PE2 and PE3 in H2B-turn-on reporter 48 hours after nucleofection. a, The tdTomato positive cell populations are gated in the represented FACS plots. **b**. Bar is represented as mean ± S.D. *P* values were calculated using a Student's two-tailed t-test. Source data are provided as a Source Data file for **b**.

Supplementary Figure 2

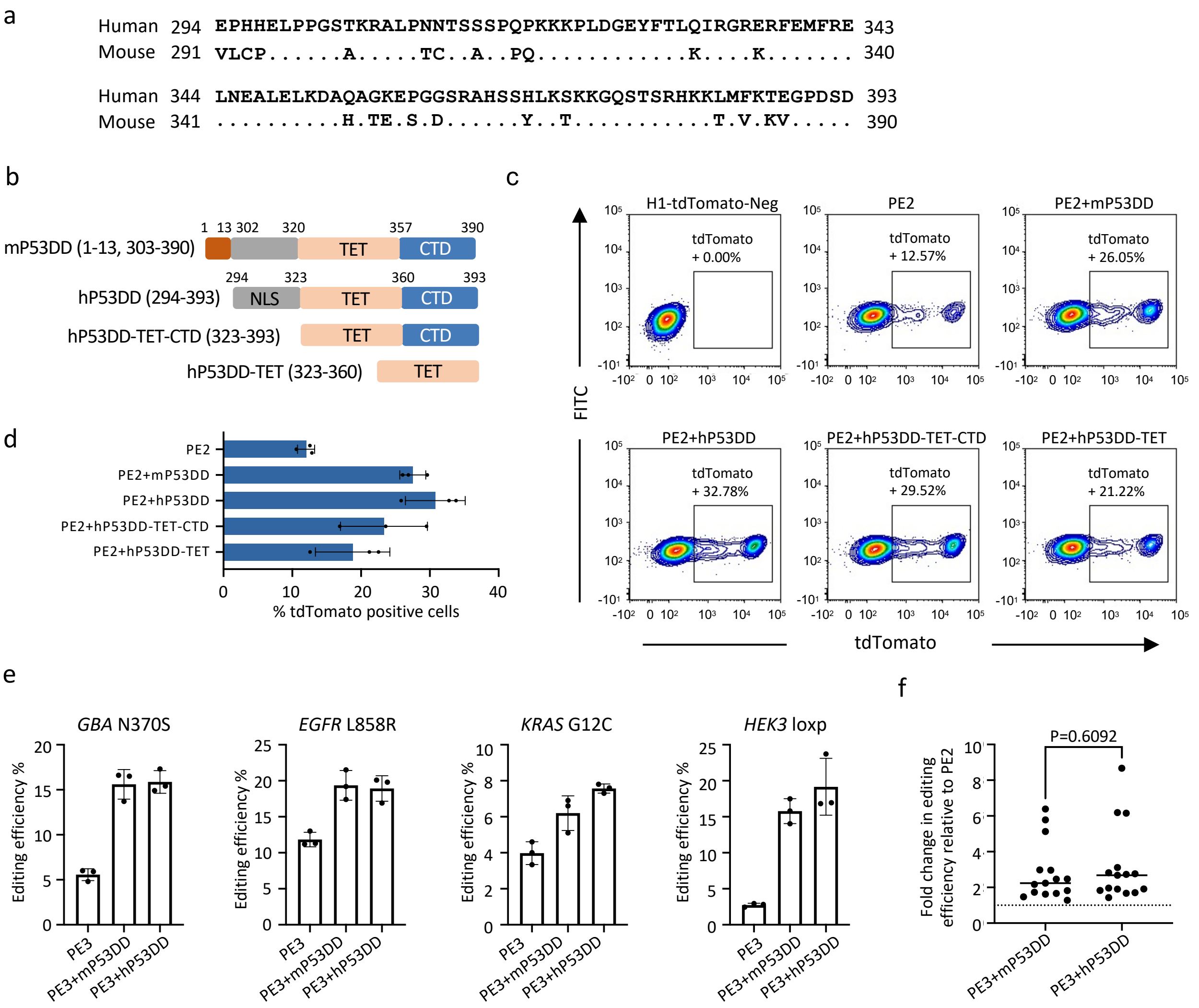

Supplementary Fig. 2: Evaluation of different versions of P53DD for the improvement of prime editing. a, Sequences of the C-terminal regions of human and mouse p53. Dots represent identical amino acids between the two species. **b,** Generation of human-derived P53DD by using full C-terminal region of human P53, containing NLS, TET and CTD domains, and two truncated versions consisting of TET and CTD domains or the TET domain only. **c, d,** Assessment of PE efficiency in the presence of different versions of P53DD by flow cytometry. Representative FACS plots (c) showing the percentages of tdTomato positive cells at 48h post-electroporation. The bar graph (d) represents the mean ± S.D from four independent experiments. **e.** Prime editing frequencies of PE3 and PE3 with mouse or human P53DD in the induction of disease mutations, including *GBA* N370S, *EGFR* L858R, and *KRAS* G12C, determined by ddPCR, and insertion of the LoxP sequence at the *HEK3* locus measured by MiSeq. Bars represented the mean ± S.D. of n=3 independent experiments. **f.** Comparison of the efficacy of mouse and human P53DD in the enhancement of prime editing. The editing efficiencies with mouse and human P53DD from five loci (data from Figure 2d and e) were normalized to PE3. The center line shows medians of all the data points from five genome loci and three independent experiments for each locus. *P* values were calculated using a Student's two-tailed t-test. Source data are provided as a Source Data file for **d, e, and f**.

Supplementary Figure 3

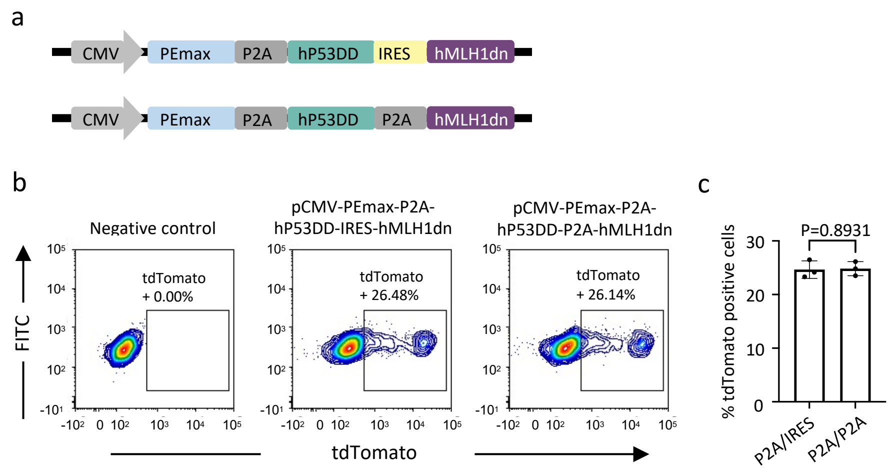

Supplementary Fig. 3: Editing efficiency of two all-in-one prime editors with different linkages for co-expression of hP53DD and hMLH1dn. Construction of two all-in-one using P2A and IRES linkages or two P2A linkages for the co-expression of PEmax, hP53DD and hMLH1dn. **b, c,** Flow cytometry analysis of the editing efficiency by the two all-in-one prime editors using H2B-turn-on reporter. The gated tdTomato positive cell populations are showed in the representative FACS plots (b), and bar graph (c) from three independent experiments. Data is represented as mean ± S.D. *P* values were calculated using a Student's two-tailed t-test (ns, non-significant). Source data are provided as a Source Data file for **c**.

Supplementary Figure.4

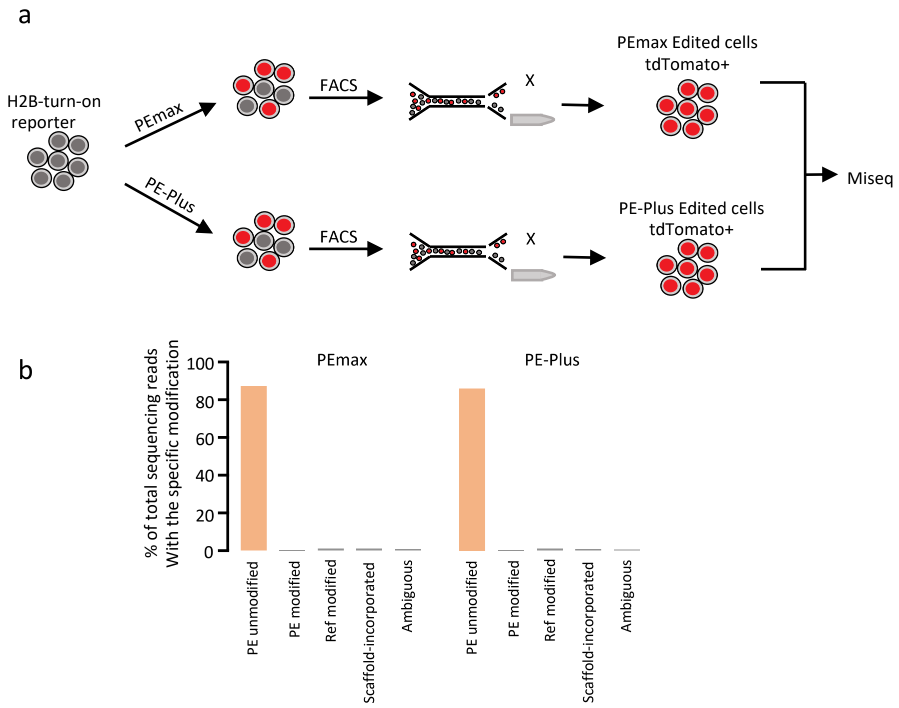

Supplementary Fig. 4: **Miseq analysis of sorted tdTomato-positive cells activated by PEmax and PE-Plus. a.** Schematic of tdTomato-positive cell isolation for Miseq analysis. FACS sorting was conducted 48 hours after the H2B-turn-on reporter was edited with PEmax or PE-Plus. DNA was collected for Miseq analysis. **b.** Intended editing (PE unmodified) and various of by-products determined by Miseq in the FASC isolated cells. Source data are provided as a Source Data file for **b**.

Supplementary Figure.5

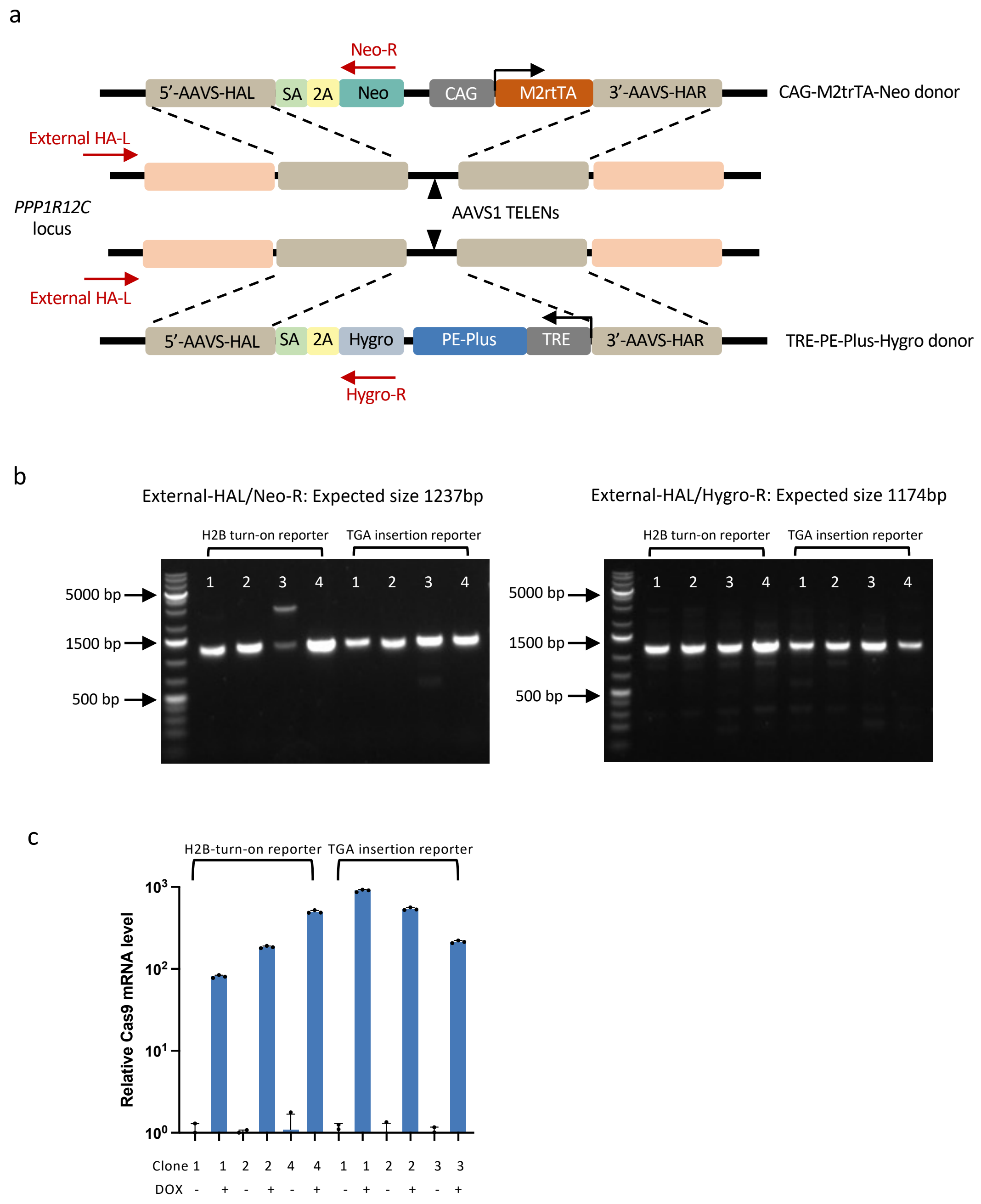

Supplementary Fig. 5: Engineering an iPE-Plus hPSC Line for Inducible Prime Editing. a. Schematic of the generation of iPE-Plus hPSCs using TALEN-mediated gene targeting at the AAVS1 site. One donor plasmid (CAG-M2trTA-Neo donor) carrying the doxycycline-controlled reverse tetracycline transactivator (M2rtTA) driven by a constitutive promoter (CAG) and another donor plasmid (TRE-PE-Plus-Hygro donor) containing a doxycycline-response element (TRE3G)-driven PE-Plus were introduced into the *PPP1R12C* intron 1 using a pair of TALEN vectors. SA, splice acceptor; Neo, Neomycin resistance gene; Hygro, Hygromycin resistance gene; HAL, homology arm left; HAR, homology arm right. Primers used for genotyping the single-cell clones are indicated by red arrows. **b.** Genotyping of single-cell clones by PCR amplifications of the region spanning the TALEN recognition site. Gel images show PCR amplicons obtained with two pairs of primers to verify the insertion of two donors at the AAVS1 site in the H2B-turn-on reporter and TGA-insertion reporter cells. **c.** Quantitative RT-PCR analysis of Cas9 mRNA levels before and after doxycycline treatment in three iPE-Plus single-cell clones generated on top of two reporter cells. Data represent the mean ± S.D. from three replicates. Source data are provided as a Source Data file for **c**.

Supplementary Figure.6

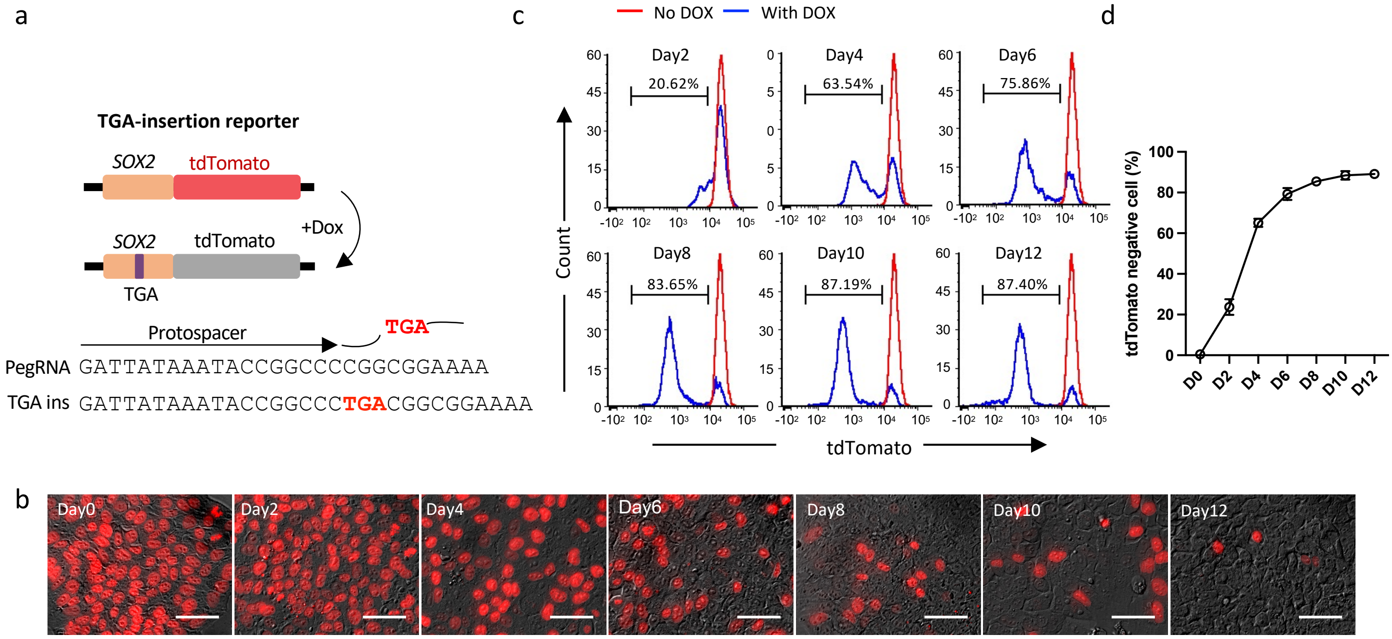

Supplementary Fig. 6: Inducible stop codon insertion into endogenous *SOX2* locus. a, Schematic of an inducible insertion of "TGA" into *SOX2* to silence tdTomato in-frame with *SOX2*. **b,** Fluorescence images showing tdTomato silencing using iPE-Plus platform in the TGA-insertion reporter after 0 to 12 days of doxycycline treatment. Representative images from four independent experiments are shown. **c, d,** Representative histograms (c) and summary plot (d) of the percentage of tdTomato-negative cells with iPE-Plus platform at indicated time points of doxycycline treatment. Untreated (red), doxycycline treated (blue). Data represents the mean ± S.D. from four replicants. Source data are provided as a Source Data file for **d**.

Supplementary Figure.7

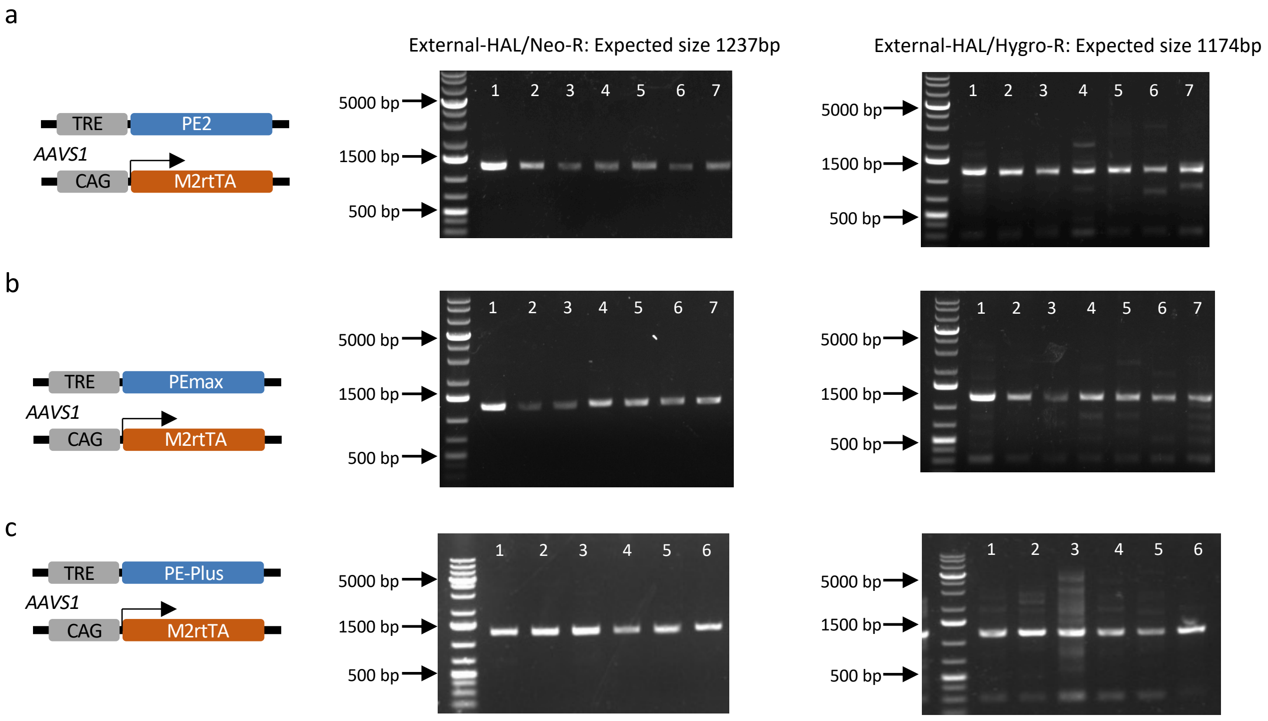

Supplementary Fig. 7: Generation of iPE, iPEmax, iPE-Plus system in hPSC H1 cells. The inducible prime editor platforms including iPE (a), iPEmax (b) and iPE-Plus (c) were engineered by knock-in at the *AAVS1* site with the corresponding prime editor driven by the TRE promoter in one allele locus and M2rtTA under the CAG promoter in another allele. Left panel: Scheme of inducible prime editor platform; Right two panels: PCR verification of knock-in of the two donors.

Supplementary Figure.8

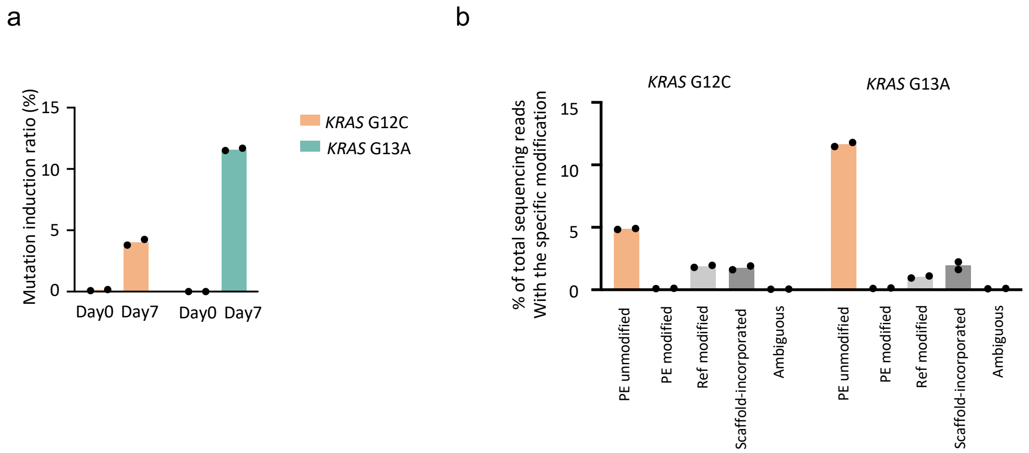

Supplementary Fig. 8: Induction of G12C and G13A mutations using iPE-Plus platform. a, Evaluation of the *KRAS* G12C and *KRAS* G13A mutation rates at different time points within one week, determined by ddPCR. **b,** Mi-seq analysis of intended editing (PE unmodified) and by-products of G12C and G13A mutations in the *KRAS* gene using iPE-Plus at day 7. Bars represent the mean from two single-cell clones. Source data are provided as a Source Data file.

Supplementary Figure.9

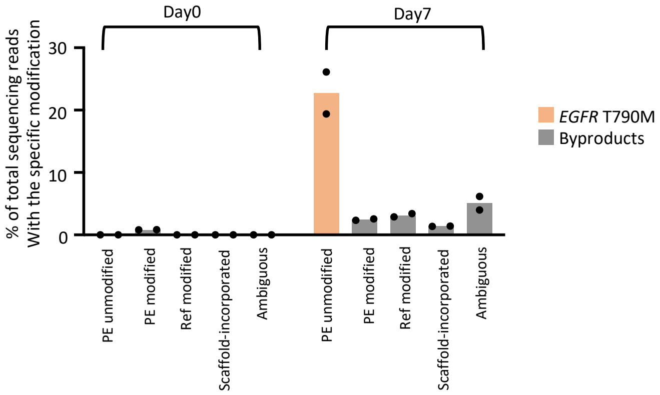

Supplementary Fig. 9: Induction of T790M mutation in EGFR gene with iPE-Plus. The intended editing (PE unmodified) and unwanted byproducts were measured by Miseq at day 0 and day 7 with DOX induction. Bars represent the mean from two single-cell clones. Source data are provided as a Source Data file.

Supplementary Figure. 10

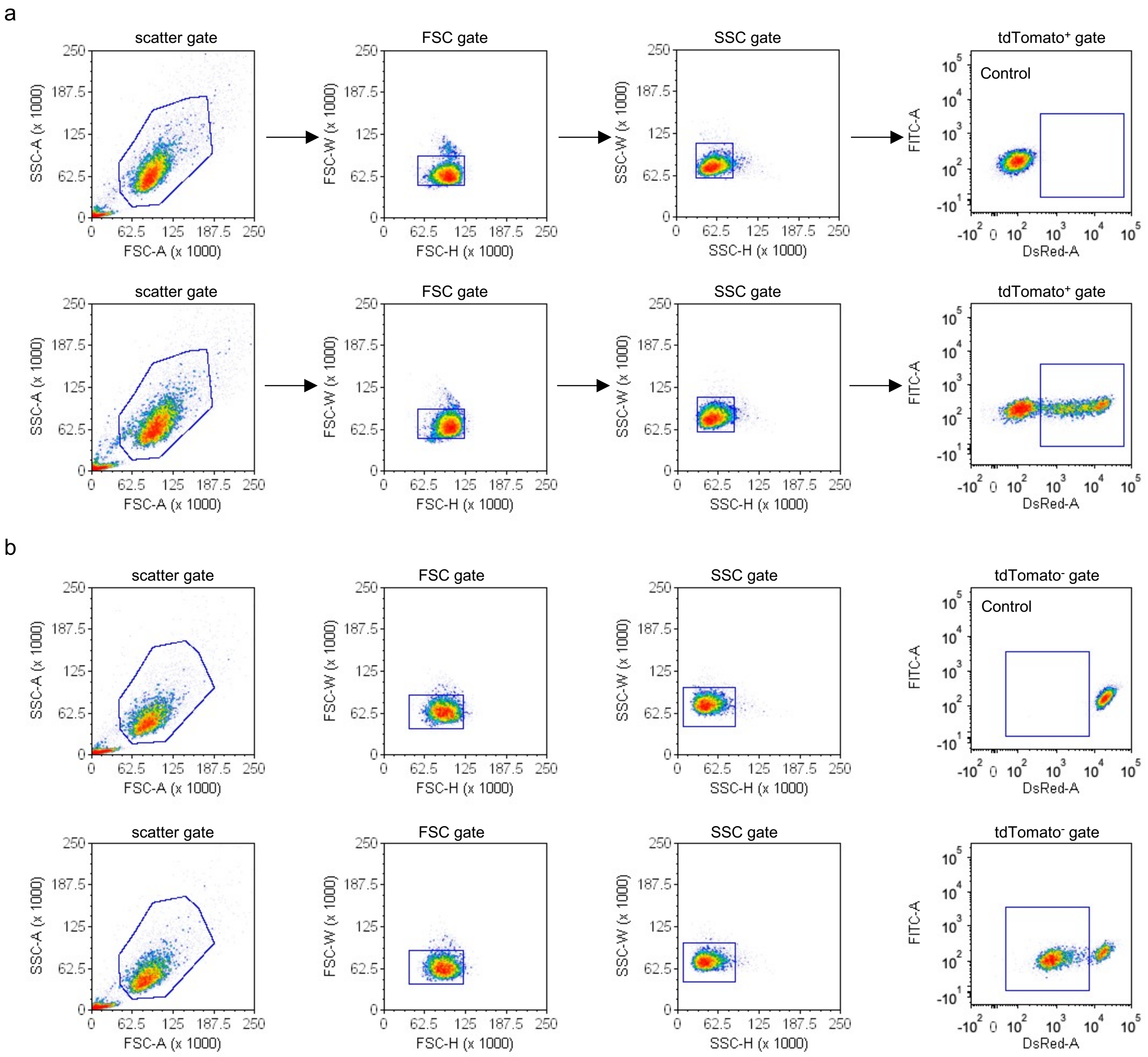

Supplementary Fig. 10: FACS gating examples for tdTomato reporter assays. Cells were initially gated on population using FSC-A/SSC-A (scatter gate). Single cells were gated using FSC-W/FSC-H (FSC gate) and SSC-W/SSC-H (SSC gate). **a**. For measuring PE editing efficiency using a H2B-turn-on reporter, the untreated reporter cells (tdTomato negative) were used as a control for gating the tdTomato positive cell population with prime editing. Data presented in Figure 1-3, Supplementary Figure 1, 2c, 2d, 3. **b.** For measuring PE editing efficiency using inducible insertion of “TGA” into SOX2 to silence tdTomato in-frame with SOX2, the inducible cell line without DOX treatment (tdTomato positive) were used for gating the tdTomato negative cell population with prime editing. Data presented in Supplementary Figure 6.

Supplementary Figure. 11

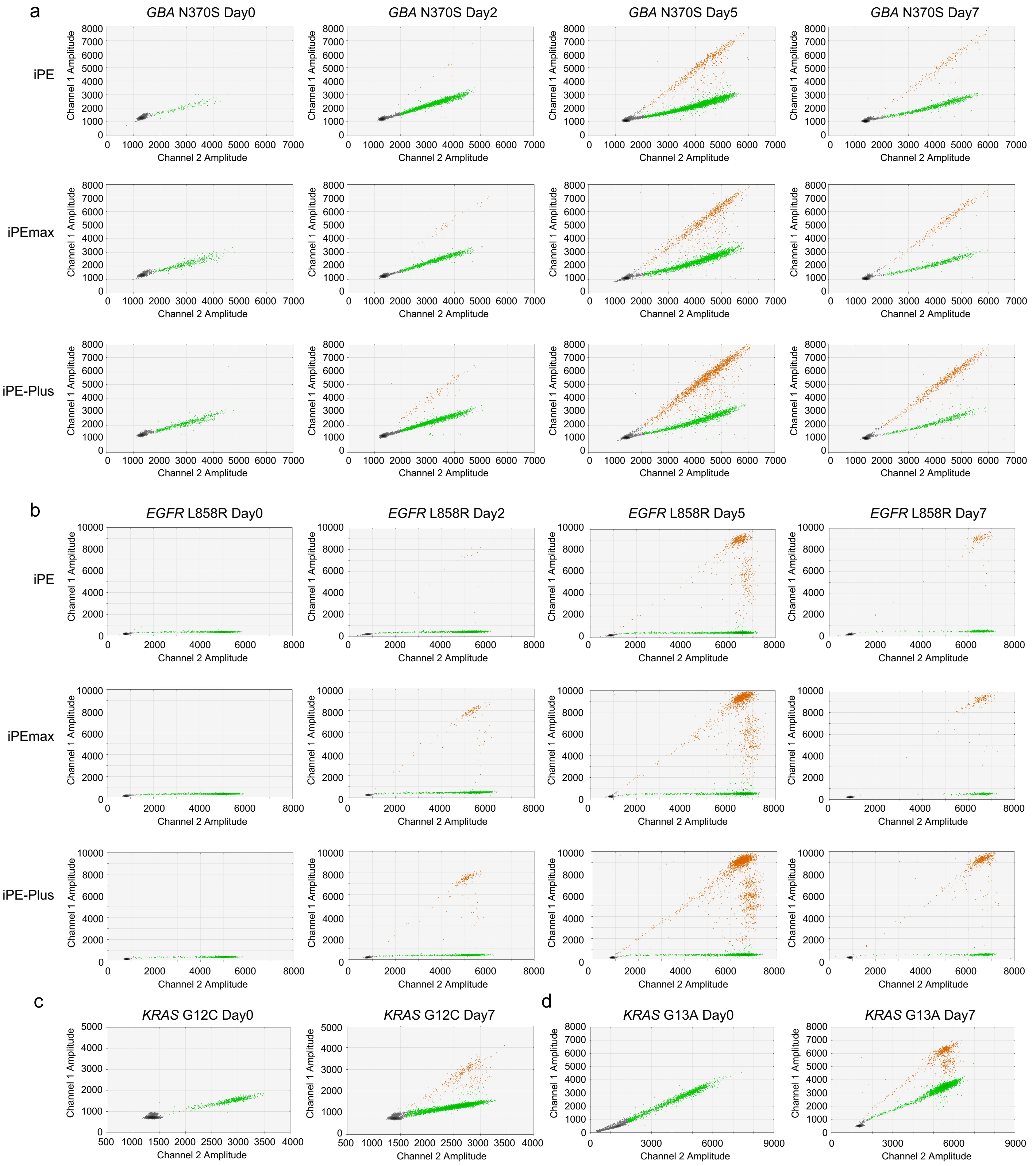

Supplementary Fig. 11

e

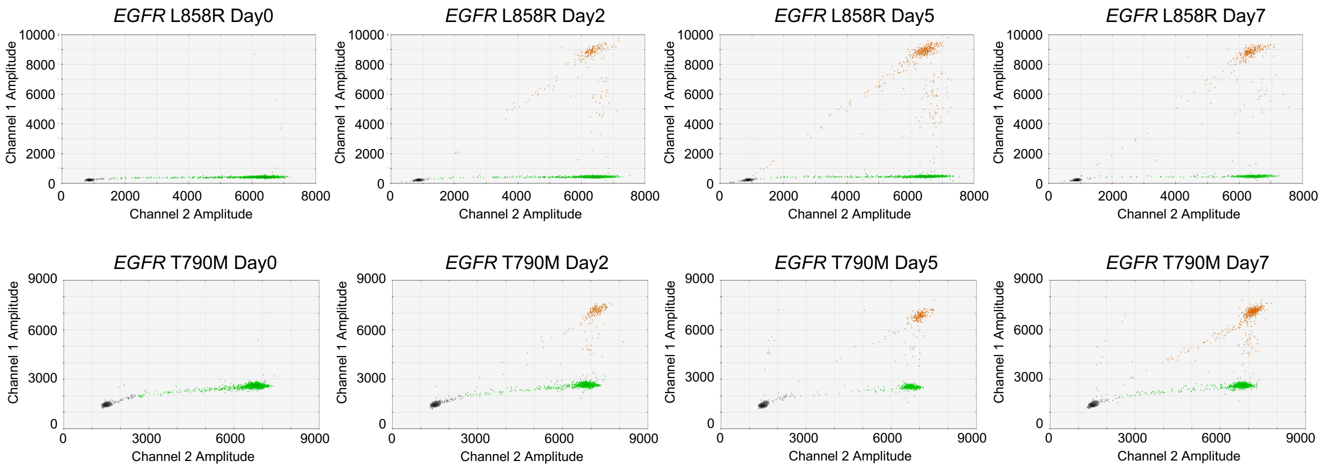

Supplementary Fig. 11: Evaluation of disease-related mutation rates by ddPCR. a, b, Representative 2D plots showing *GBA* N370S mutation rates (a) and *EGFR* L858R mutation (b) at indicated time points with doxycycline treatment. **c, d,** Representative 2D plots showing G12C mutation (c) and G13A mutation (d) in the *GBA* gene after 7 days of induction with the iPE-Plus platform. **e,** One-step dual mutations in the *EGFR* gene induced by the iPE-Plus platform, determined by ddPCR.

Supplementary Table 1. pegRNAs and nicking sgRNA sequences

pegRNA target site	Editing type	intended edit	pegRNA construct	pegRNA spacer (5'-3')	3' extension sequence (5'-3')	nicking sgRNA spacer	Reference
SOX2	Insertion	"TGA" insertion	lenti-epegRNA-SOX2-TGA	ATTATAAATACCGGCCCCGG	GTTTTCCGCCGTCAGGGCCGGTATTTAT	N/A	Supplementary Fig. 6
GBA	Substitution	c.1226 A>G	lenti-epegRNA-GBA-N370S; epegRNA-GBA-N370S	AGCCGACCACATGGTACAGG	TTACCCTAGAGCCTCCTGTACCATGTGGTC	ACCCTTACCTACACTCTCTG	Fig. 4e; Supplementary Fig. 2e, f
LRRK2	Substitution	c.6055 G>A	lenti-epegRNA-LRRK2-G2019S	ATTGCAAAGATTG CTGACTA	AGCAATGCTGTAGTCAGCAATCTTTGC	GACAGACCTGATCACCTACC	Fig. 4h
EGFR	Substitution	c.2573T>G	lenti-epegRNA-EGFR-L858R; epegRNA-EGFR-L858R	CAAGATCACAGATTTTGGGC	AGTTTGGCCCGCCCAAAATCTGTGATCT	TTACTTTGCCTCCTTCTGCA	Fig. 4f; Supplementary Fig. 2e, f
EGFR	Substitution	c.2369C>T	lenti-epegRNA-EGFR-T790M	TAGTCCAGGAGGCAGCCGAA	ATCATGCAGCTCATGCCCTTCGGCTGCCTCCTG	CCTCCAGGAAGCCTACGTGA	Supplementary Fig. 9
EGFR	Substitution	c.2573T>G /c.2369C>T	lenti-epegRNA-EGFR-L858R-T790M	CAAGATCACAGATTTTGGGC TAGTCCAGGAGGCAGCCGAA	AGTTTGGCCCGCCCAAAATCTGTGATCT ATCATGCAGCTCATGCCCTTCGGCTGCCTCCTG	TTACTTTGCCTCCTTCTGCA CCTCCAGGAAGCCTACGTGA	Fig. 6
KRAS	Substitution	c.34G>T	lenti-epegRNA-KRAS-G12C; epegRNA-KRAS-G12C	CTTGTGGTAGTTGGAGCTGG	TGCCTACGCCACAAGCTCCAACTACCA	ACAAGATTTACCTCTATTGT	Supplementary Fig. 8; Supplementary Fig. 2e, f
KRAS	Substitution	c.38G>A	lenti-epegRNA-KRAS-G13A	CTTGTGGTAGTTGGAGCTGG	TGCCTACGTCACCAGCTCCAACTACCA	ACAAGATTTACCTCTATTGT	Supplementary Fig. 8
HEK3	Insertion	34nt LoxP insertion	pegRNA-HEK3-loxp; lenti-epegRNA-HEK3-loxp	GGCCCAGACTGAGCACGTGA	TTTGCCATCAATAACTTCGTATAATGTATGCTATACGAAGTTATCGTGCTCAGTCTG	GTCAACCAGTATCCCGGTGC	Fig. 1l; Fig. 4i; Supplementary Fig. 2e, f
HEK3	deletion	2nt deletion	pegRNA-HEK3-2nt-del	GGCCCAGACTGAGCACGTGA	TCCTTTGCCATCGTGCTCAGTCTG	GTCAACCAGTATCCCGGTGC	Fig. 1j
HEK3	deletion	30nt deletion	pegRNA-HEK3-30nt-del	GGCCCAGACTGAGCACGTGA	TGTCCTGCGACGCCCTCTGGACGTGCTCAGTCTG	GTCAACCAGTATCCCGGTGC	Fig. 1k
SOX2	deletion	10nt deletion	epegRNA-SOX2-10nt-del	ATTATAAATACCGGCCCCGG	TGAGCGTCTTGGGGGCCGGTATTT	CGCTTAGCCTCGTCGATGAA	Fig. 1m
SOX2	deletion	40nt deletion	epegRNA-SOX2-40nt-del-twinPE-A; epegRNA-SOX2-40nt-del-twinPE-B	ATTATAAATACCGGCCCCGG; GCTCGCCATGCTATTGCCGC	TTCTTCATGAGCGTCTTGGTTTTCCGCCGGGGCCGGTATTT; GGCGGAAAACCAAGACGCTCATGAAGAAGGCAATAGCATGGC	N/A	Fig. 1n

Supplementary Table 2. Sequences of primers and probes used for ddPCR assays

Target	Forward primer	Reverse primer	Edit probe	Reference probe
GBA N370S c.1226 A>G	TGTTGCGCCTTTGTCTCT	GATGGGACTGTCGACAAAGTTA	/56-FAM/ACCCTAGAG/ZEN/CCTCCTGTACCA/3IABkFQ/	/5HEX/AACCTTGCC/ZEN/CTGAACCCCGA/3IABkFQ/
EGFR L858R c.2573T>G	CTTTCTCTTCCGCACCCA	GTCTTCTCTGTTTCAGGGCAT	/56-FAM/TTTGGCCCG/ZEN/CCCAAAATCT/3IABkFQ/	/5HEX/TCGCGGTGC/ZEN/ACCAAGCGAC/3IABkFQ/
EGFR T790M c.2369C>T	TCCAGGAAGCCTACGTGAT	CTTTGTGTTCCCGGACATAGT	/56-FAM/ATGAGCT+G+CA+T+GATGAG/3IABkFQ/	/5HEX/CTCA+CCTCC+A+CCGTGC/3IABkFQ/
KRAS G12C c.34G>T	CTGTATCGTCAAGGCACTCT	AGGTACTGGTGGAGTATTTGATAG	/56-FAM/CGCCACAAG/ZEN/CTCCAACTACCACAA/3IABkFQ/	/5HEX/ATTCAGTCA/ZEN/TTTTCAGCAGGCCTTA/3IABkFQ/
KRAS G13A c.38G>A	CTGTATCGTCAAGGCACTCT	AGGTACTGGTGGAGTATTTGATAG	/56-FAM/CGTCACCAG/ZEN/CTCCAACTACCACAA/3IABkFQ/	/5HEX/ATTCAGTCA/ZEN/TTTTCAGCAGGCCTTA/3IABkFQ/

Supplementary Table 3. Primers for Miseq

Target	Forward primer	Reverse primer
GBA N370S c.1226 A>G	CCTTGAGATGCCTGGATCTTC	CGACAAAGTTACGCACCCAAT
EGFR L858R c.2573T>G	TCAGTAGTCACTAACGTTCGCCAGC	AGCTCACCCAGAATGTCTGGAG
EGFR T790M c.2369C>T	TCGCATTCATGCGTCTTCACCTG	AACTCTTGCTATCCCAGGAGCG
KRAS G12C c.34G>T	ACTTGAAACCCAAGGTACATTTCAG	AGTGTATTAACCTTATGTGTGACATGTTCT
KRAS G13A c.38G>A	ACTTGAAACCCAAGGTACATTTCAG	AGTGTATTAACCTTATGTGTGACATGTTCT
H2B	TTAACGGCACACTGCCCCTCTCA	TGGCCTTAGTACCCTCGGACACG
SOX2	GGACCGCGTCAAGCGGC	TCATGCTGTAGCTGCCGTTGC
HEK3	CTTGGCATGAGAAACCTTGG	TCCCTCCTCTCCTGGTGAG
LRRK2	TAAGGGACAAAGTGAGCACAG	CACATCTGAGGTCAGTGGTTATC

Supplementary Table 4. Primers for single-cell clone genotyping

Target	Amplicon primer (forward)	Amplicon primer (Reverse)	Sanger sequencing primer
AAVS1 KI (CAG-M2trTA-Neo donor)	External-HAL:ACCTACTCTCTTCCGCATTGG	Neo-R: CTCGTCCTGCAGTTCATTCA	ATTCAGGGCACCGGACAGGT
AAVS1 KI (TRE-prime editor-Hygro)	External-HAL:ACCTACTCTCTTCCGCATTGG	Hygro-R: GACATATCCACGCCCTCCTA	CTGCCGTCTCTCTCCTGAGT
GBA N370S c.1226 A>G	CCTTGAGATGCCTGGATCTTC	CGACAAAGTTACGCACCCAAT	CGACAAAGTTACGCACCCAAT
EGFR L858R c.2573T>G	TCAGTAGTCACTAACGTTCGCCAGC	AGCTCACCCAGAATGTCTGGAG	TCAGAGCCTGGCATGAACATGAC
EGFR T790M c.2369C>T	TCGCATTCATGCGTCTTCACCTG	AACTCTTGCTATCCCAGGAGCG	TGTGAGGATCCTGGCTCCTTATC
