# Supplementary material for: Robust and inducible genome editing via an all-in-one prime editor in human pluripotent stem cells
Source: Nat Commun. 2024 Dec 30;15:10824. doi: 10.1038/s41467-024-55104-1 (PMC11685797; doi:10.1038/s41467-024-55104-1)
Supplement: Supplementary file 2 — Reporting Summary [file 41467_2024_55104_MOESM2_ESM.pdf]

nature portfolio

Corresponding author(s): Lorenz Studer and Ting Zhou

Last updated by author(s): Oct 21, 2024

Reporting Summary

Nature Portfolio wishes to improve the reproducibility of the work that we publish. This form provides structure for consistency and transparency in reporting. For further information on Nature Portfolio policies, see our Editorial Policies and the Editorial Policy Checklist.

Statistics

For all statistical analyses, confirm that the following items are present in the figure legend, table legend, main text, or Methods section.

n/a	Confirmed	
☐	☒	The exact sample size (*n*) for each experimental group/condition, given as a discrete number and unit of measurement
☐	☒	A statement on whether measurements were taken from distinct samples or whether the same sample was measured repeatedly
☐	☒	The statistical test(s) used AND whether they are one- or two-sided<br>*Only common tests should be described solely by name; describe more complex techniques in the Methods section.*
☒	☐	A description of all covariates tested
☒	☐	A description of any assumptions or corrections, such as tests of normality and adjustment for multiple comparisons
☐	☒	A full description of the statistical parameters including central tendency (e.g. means) or other basic estimates (e.g. regression coefficient) AND variation (e.g. standard deviation) or associated estimates of uncertainty (e.g. confidence intervals)
☐	☒	For null hypothesis testing, the test statistic (e.g. *F*, *t*, *r*) with confidence intervals, effect sizes, degrees of freedom and *P* value noted<br>*Give P values as exact values whenever suitable.*
☒	☐	For Bayesian analysis, information on the choice of priors and Markov chain Monte Carlo settings
☒	☐	For hierarchical and complex designs, identification of the appropriate level for tests and full reporting of outcomes
☒	☐	Estimates of effect sizes (e.g. Cohen's *d*, Pearson's *r*), indicating how they were calculated

Our web collection on statistics for biologists contains articles on many of the points above.

Software and code

Policy information about availability of computer code

Data collection	Flow cytometry was conducted using BD FACSAria III instrument. Sanger sequencing data was collected by submitting PCR product to Eton Biosciences. Miseq data was collect by submitting purified PCR product to Genewiz for Amplicon sequencing (Amplicon EZ service, illumina Miseq system). Droplet digital PCR data was collected using QuantaSoft (version 1.4, Bio-Rad).
Data analysis	Flow cytometry data was analyzed using FCS Express software (version 7.18.0025, DeNovo Software). Miseq data was analyzed by alignment of amplicon sequences to a reference sequence using open web resource CRISPRess2: (http://crispresso2.pinellolab.org/submission).Droplet digital PCR data was analyzed using QuantaSoft (version 1.4, Bio-Rad). The statistical analyses were performed using GraphPad Prism 9.

For manuscripts utilizing custom algorithms or software that are central to the research but not yet described in published literature, software must be made available to editors and reviewers. We strongly encourage code deposition in a community repository (e.g. GitHub). See the Nature Portfolio guidelines for submitting code & software for further information.

Data

Policy information about availability of data

All manuscripts must include a data availability statement. This statement should provide the following information, where applicable:

- Accession codes, unique identifiers, or web links for publicly available datasets
- A description of any restrictions on data availability
- For clinical datasets or third party data, please ensure that the statement adheres to our policy

Miseq data and WGS data are deposited in the NCBI BioProject PRJNA1047080.

Research involving human participants, their data, or biological material

Policy information about studies with human participants or human data. See also policy information about sex, gender (identity/presentation), and sexual orientation and race, ethnicity and racism.

Reporting on sex and gender	NA
Reporting on race, ethnicity, or other socially relevant groupings	NA
Population characteristics	NA
Recruitment	NA
Ethics oversight	NA

Note that full information on the approval of the study protocol must also be provided in the manuscript.

Field-specific reporting

Please select the one below that is the best fit for your research. If you are not sure, read the appropriate sections before making your selection.

☒ Life sciences ☐ Behavioural & social sciences ☐ Ecological, evolutionary & environmental sciences

For a reference copy of the document with all sections, see nature.com/documents/nr-reporting-summary-flat.pdf

Life sciences study design

All studies must disclose on these points even when the disclosure is negative.

Sample size	Statistical analyses were performed with three or more than three independent experimental replicates. Sample sizes, statistical methods and p values were provided in each figure legend as well as the Source Data. For the experiments using inducible PE single cell clones (figure 4b-c, 4e-f, 4h-i, 6b-c), two independent single-cell clones for each type of inducible line were tested to avoid single-cell clone variation.
Data exclusions	No data were excluded from the analyses.
Replication	All other experiments were performed at least two biological replicates. All the attempts at replication were successful.
Randomization	No randomization was performed. Cell culture conditions from all independent biological replicates were treated identical.
Blinding	Generally no blinding was performed as data are not subjective. Specifically, Miseq and Sanger sequencing were performed by 3rd party unaware of the treatment conditions. The gating of Flow cytometry analysis was set up based on negative control, and those gating conditions were applied to all samples.

Reporting for specific materials, systems and methods

We require information from authors about some types of materials, experimental systems and methods used in many studies. Here, indicate whether each material, system or method listed is relevant to your study. If you are not sure if a list item applies to your research, read the appropriate section before selecting a response.

Materials & experimental systems

n/a	Involved in the study
☐	☒ Antibodies
☐	☒ Eukaryotic cell lines
☒	☐ Palaeontology and archaeology
☒	☐ Animals and other organisms
☒	☐ Clinical data
☒	☐ Dual use research of concern
☒	☐ Plants

Methods

n/a	Involved in the study
☒	☐ ChIP-seq
☐	☒ Flow cytometry
☒	☐ MRI-based neuroimaging

Antibodies

Antibodies used	BD Pharmingen™ Purified Mouse anti-Human Pax-6, Clone O18-1330 (RUO), Catalog No:561462
Validation	Supporting documentation, references, and validation statements are available at the manufacturer's website.

Eukaryotic cell lines

Policy information about cell lines and Sex and Gender in Research

Cell line source(s)	H1 hESCs were purchased from WiCell Institute. The H1-SOX2-tdTomato reporter line was generated by knocking in P2A-H2B-tdTomato cassette before the stop codon at the SOX2 locus through CRISPR-mediated HDR in H1 cells. The H2B-tdTomato turn-on reporter line was generated by knocking in a mutated P2A-H2B-tdTomato cassette before the stop codon at the SOX2 locus through CRISPR-mediated HDR in H1 cells. The mutated P2A-H2B-tdTomato cassette carried a “C” deletion in the H2B sequence. Both reporter lines harbored heterozygous insertions of the cassette. The iPE2, iPEmax and iPE-Plus lines with inducible expression of the three prime editors were generated by introducing the donor plasmids containing the prime editor under the TRE-tight promoter, the Neo-M2rtTA donor and a pair of TALENs through co-electroporation into hPSCs, targeting the first intron of the PPP1R12C gene.
Authentication	H1 hESCs, H1-SOX2-tdTomato reporter, H2B-tdTomato turn-on reporter, iPE2, iPEmax and iPE-Plus lines were characterized with >99% pluripotent markers (OCT4/SOX2/NANOG) expression.
Mycoplasma contamination	All cell lines tested negative for mycoplasma.
Commonly misidentified lines (See ICLAC register)	All the hESC lines have been fully characterized, and are not the commonly misidentified lines.

Plants

Seed stocks	NA
Novel plant genotypes	NA
Authentication	NA

Flow Cytometry

Plots

Confirm that:

☒ The axis labels state the marker and fluorochrome used (e.g. CD4-FITC).

☒ The axis scales are clearly visible. Include numbers along axes only for bottom left plot of group (a 'group' is an analysis of identical markers).

☒ All plots are contour plots with outliers or pseudocolor plots.

☒ A numerical value for number of cells or percentage (with statistics) is provided.

Methodology

Sample preparation	Prior to FACS analysis, cells were detached using Accutase (Innovative Cell Tech. AT104) at 3TC for 10 min, followed by an addition of Stemflex Medium and centrifugation at 120 g for 3 min. Cell pellets were resuspended in 300 µl Stemflex, filtered through a Falcon 5 ml Round Bottom Polystyrene Test Tube with Cell Strainer Snap Cap (Fisher Scientific 352235), and kept on ice.
Instrument	BD FACSAria III
Software	FCS Express software (version 7.18.0025, DeNovo Software)
Cell population abundance	The tdTomato fluorescence signal was checked under microscope first before FACS analysis. Live hPSCs cells were gated based on forward and side scatter for the following analysis.
Gating strategy	Live cells were selected (side scatter area (SSC-A) versus forward scatter area (FSC-A)), followed by selection of single cells (forward scatter width (FSC-W) versus height (FSC-H) then side scatter width (SSC-W) versus side scatter height (SSC-H)).tdTomato positive or tdTomato negative cell population were gated according to the untreated control. The gating strategy is exemplified in Supplementary Figure 2.

☒ Tick this box to confirm that a figure exemplifying the gating strategy is provided in the Supplementary Information.
